# Artificial General Intelligence: Could It Suffer From a Mental Illness?

**DOI:** 10.1192/bjo.2025.10114

**Published:** 2025-06-20

**Authors:** Alistair Clarke

**Affiliations:** Cambridge University Hospitals, Cambridge, United Kingdom

## Abstract

**Aims:** Could a hypothetical future Artificial General Intelligence (AGI) suffer from a mental illness? While this question may evoke differing intuitions, the following arguments propose that such an AGI could indeed experience mental pathology.

**Methods:** To prove that an AGI could suffer from a mental illness, the method of philosophical thought experiment using a priori deductive reasoning has been employed. The argument’s premises are justified by known principles of computer science and psychiatry.

**Results:** Though AGI systems do not yet exist, exploring their potential nature can offer valuable insights into conceptualising the pathogenesis of psychiatric illness. Consider the following deductive inference: **Premise 1**: People can suffer from mental illness. **Premise 2**: A future AGI will be a person, i.e. a conscious entity capable of generating new knowledge. **Conclusion:** An AGI will be a person and therefore can suffer from a mental illness.

**Conclusion:** From computer science and physics, we know in principle that AGI must be possible. The intuition that an AGI would be conscious, and therefore susceptible to mental illness, finds support from the Church–Turing–Deutsch principle. That is to say, any Turing-complete system, which includes all modern computers, can simulate any physical system, including the human brain. The human brain is a known structure that supports general intelligence, minds and consciousness. While brains are not isolated systems and have internal and external environmental inputs and outputs, these could also be computationally simulated. The key question is whether such a simulated brain would actually be conscious or merely simulate consciousness. It seems logically incoherent to “simulate” consciousness, as a successful attempt to simulate a conscious brain would necessarily result in the creation of a conscious being. Therefore, like a human mind, it seems consistent to suggest the mind of an AGI can suffer from mental illness.

Unlike humans, an AGI will not have a biological brain. Instead, its mind will presumably run on a silicon-based substrate. This suggests that brains are not fundamental to the pathophysiology of mental illness. Rather, we can speculate that information and its aberrant processing play a more central role in the emergence of mental disorders.

